# Large Terrestrial Bird Adapting Behavior in an Urbanized Zone

**DOI:** 10.3390/ani9060351

**Published:** 2019-06-13

**Authors:** Eduardo R. Alexandrino, Juliano A. Bogoni, Ana B. Navarro, Alex A. A. Bovo, Rafael M. Gonçalves, Jacob D. Charters, Juan A. Domini, Katia M. P. M. B. Ferraz

**Affiliations:** University of São Paulo, Luiz de Queiroz College of Agriculture, Forest Sciences Department, Wildlife Ecology, Management and Conservation Lab (LEMaC). Pádua Dias, Av., P.O. Box 09, Piracicaba/SP 13418-900, Brazil; bogoni@usp.br (J.A.B.); ana.navarro@usp.br (A.B.N.); alex_bovo@hotmail.com (A.A.A.B.); rafaelmenezes@usp.br (R.M.G.); jake.d.charters@usp.br (J.D.C.); juan.domini@usp.br (J.A.D.); katia.ferraz@usp.br (K.M.P.M.B.F.)

**Keywords:** collaborative citizen science, participatory science, human-modified landscape, home range, ornithology, bird banding, bird re-sighting, avian ecology, human–wildlife conflicts, urban wildlife

## Abstract

**Simple Summary:**

As the world becomes increasingly urbanized and encroaches on natural environments, wildlife face pressure to adapt to human activities. Understanding the adaptation processes of wildlife living in urban areas is an important step in the implementation of management decisions and regulatory policies, which should aim to minimise human–wildlife conflicts in cities. We investigated a rare case of the Red-legged Seriema, a large-sized terrestrial bird, occurring in an urbanized zone in a Neotropical city. We described their behaviors and assessed their distribution based on hundreds of data provided by citizen scientists. We discovered that Seriemas are occurring in the same space occupied by many free-ranging cats within the study area, which are being supported by humans offering food provisions. Humans are also providing food for Seriemas directly. The species is also benefiting from using human-made structures to improve their behavior related to territory defense and opportunistic foraging. However, some are still unable to avoid car collisions, which is a threat to their persistence in this area. Our study suggests that humans may be contributing to the domestication process of Seriemas, which may lead to them losing fear of humans, but not necessarily acquiring behavior that is advantageous to survival in cities.

**Abstract:**

Wildlife living within urban ecosystems have to adapt or perish. Red-legged Seriema, a large terrestrial bird, are rare in urban ecosystems, however, they have been reported in a medium-sized Brazilian city. We investigated the reasons for this occurrence as well as their behavior. We assessed the distribution of Seriemas (including fledglings), free-ranging cats, and cat-feeding points provided by humans, and past records of Seriemas in the study area. We discovered that Seriemas are sharing spatial resources with cats without apparent conflicts, and intraspecific competition was important to define the spatial distribution of Seriemas. This species is able to use human-made structures to improve territory defense and opportunistic foraging. Direct and indirect human food provisioning is helping them to survive in the studied area, but is also facilitating the domestication process, which may cause future conflicts with humans and cats. Although Seriemas have inhabited the studied urban area for years, they are still adapting their behaviors for urban life, as they have not yet perceived the dangers of automotive traffic. Our study corroborates that wild species may adapt to urban areas driven by human contact, but it also acts as a trap for the adaptive process.

## 1. Introduction

Urbanized zones are considered unique and complex ecosystems [1,2] and the process of urbanization has been shown to exert drastic changes on the natural landscapes [3]. The complexity within urbanized zones is mainly a consequence of human activities, which are based on social, cultural, and political aspects, driving rapid and dynamic changes in the urban structure [4,5]. Understanding adaptation processes of wildlife in urban zones is an important step for making and implementing management decisions and regulatory policies, with the aim of avoiding future human–wildlife conflicts in cities [6].

In eco-friendly urban areas (i.e., sparsely populated zones or green areas, such as parks or peri-urban areas) the presence of native wildlife, particularly generalist species, may be relatively high (e.g., [7,8,9]) and encounters with humans are common. Some wild species, such as mammals and birds, may be considered charismatic [10,11], and people visiting these areas may be inclined to interact with them or provide food [12,13]. Although this activity may seem harmless, there is empirical evidence that shows that there can be detrimental effects, such as excessive increases in body mass (e.g., Silver Gull, [14]) and increased fighting among individuals (e.g., [15]). Further, wildlife occurring in urban areas are more prone to collide with human-made structures [7,16,17], become victims of car collisions [18,19,20], be negatively impacted by domestic animals [21,22], and are even forced to modify intraspecific communication in response to urban noise [23]. Thus, urban areas may test the limits of tolerance of many native species [24,25,26]. Modified or learned behavior in relation to perceiving food availability and identifying risks of death or injury are two major factors that drive this adaptive process [25]. For wildlife species living in such environments, to maximize the chances of survival these adaptations need to occur as quickly as possible, such as through rapid physiological changes (e.g., [23]).

In recent years, small groups of Red-legged Seriema (*Cariama cristata*) (hereafter Seriema), a large terrestrial bird (i.e., 90 cm height, weight up to 1.5 kg, [27]), have become common in the urbanized area of a university campus in the city of Piracicaba (“Luiz de Queiroz” campus, University of Sao Paulo), southeast Brazil (see recent records [28,29,30,31]). The species is known to be anthropogenic-tolerant [27,32] and non-threatened [33], but rarely occurs in areas of higher levels of urbanization (e.g., [8,34,35]). Aiming to provide an answer as to why and how Seriemas are living in close proximity to humans, we investigated the species occurrence and behavior of the Seriemas living on campus. Although there are many existing studies focusing on bird behavior in urban areas, most have focused on small to medium sized birds, while large terrestrial species remain largely ignored, especially in Neotropical cities [36]. Here, we collected hundreds of data points of the distribution of individual Seriemas throughout the urbanized area of the campus from citizen scientists, whilst also focusing on the distribution of free-ranging cats and human food provisions in the same space. Thus, we aimed to: (1) understand the use of space for individual Seriemas living in an urban area of the “Luiz de Queiroz” campus; (2) evaluate the shared space between Seriemas, free-ranging domestic cats, and the distribution of artificial food provisions (dry cat food); and (3) depict the behavioral biology of individual Seriemas living in this urbanized area. We hypothesized that the Seriema is able to live in this urbanized zone owing to its biological plasticity. Moreover, free-ranging cats are not a threat to Seriema due to the species’ large size, while the artificial food provision for cats can lead to a non-intentional domesticating process and increase Seriema’s survival. Finally, intraspecific competition is a more important ecological process than interspecific competition with regards to the spatial distribution of the Seriema in the current urbanized zone.

## 2. Materials and Methods

### 2.1. Study Area

**Figure 1:**
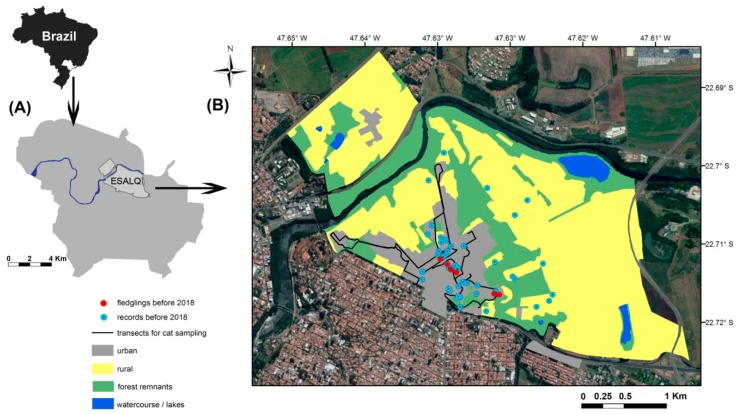
(**a**) Location of the Luiz de Queiroz campus (also known as ESALQ, the acronym for the college’s name) in the southeast Brazil. (**b**) Main land use in the campus, transects used for cat sampling in 2017, and records of Red-legged Seriema and their fledglings before 2018. Only those records of which we discovered the exact location were included in the map (details of all historical records are available in the supplementary materials, Table S1).

Our study was carried out at Luiz de Queiroz College of Agriculture (hereafter LQ campus), belonging to the University of São Paulo, located in Piracicaba, state of São Paulo, in southeastern Brazil (22°43′30″ S, 47°38′00″ W). In 1901, at the time of its foundation, the campus was a typical farm in an agricultural landscape, 3 km from the Piracicaba urban matrix [37,38]. Throughout the 20th century, the urban expansion of Piracicaba gradually converted agricultural land around the campus into urbanized zones [8]. Meanwhile, academic courses in the university also expanded, leading to the establishment of new buildings and the consolidation of an urban scheme inside the campus. Currently, LQ campus is made up of approximately 860 ha of heterogeneous landscape, composed of around 512.2 ha of agricultural land (59% of the territory, including barns, small facilities, forestry plantations), 212.1 ha of native forest patches (24.6%, including old forest and active restored patches), and 116.6 ha are urbanized zones (13.5%, including green areas, as gardens and woodlots, enjoyed by the public). There are also watercourses, and the main river of the region, Piracicaba River, along the northern edge of the campus (Figure 1). The region’s climate is categorized as Cwa (warm summer and dry in winter, [39]) and lies 546 m above sea level. It is within the Atlantic Forest biome in a region originally covered by Seasonally Tropical Dry Forest [3,40]. Students and university staff frequent the area, and the campus is a popular leisure area for residents from Piracicaba (mainly during weekends and late afternoons, e.g., for picnics, jogging and other soft sports).

### 2.2. Domestic Cat Sampling

For years, the LQ campus has suffered from the presence of free-ranging domestic cats. In 2003, 81 cats were registered on campus [21]. Since then, continued breeding among resident cats, the further releasing or abandoning of unwanted pet cats by city residents, the provision of food by well-meaning residents, and a lack of the implementation of population control programs by the campus administration has led to a further increase in the cat population on campus [9]. In 2017, we counted by ad libitum [41] all cats observed over 21 days (August to November 2017) along a 9.7 km transect on LQ campus (Figure 1). All sampling was done by the same field observer. In each sampling day, one starting point was chosen (i.e., there were three standard starting points) and the transect fully investigated. Only diurnal (7:00 up to 10:00) or evening periods (17:00 up to 22:00) were used for sampling, alternating each period in each sampling day. We conducted 11 diurnal sampling and 10 evening sampling periods. Every cat counted during the sampling was photographed to be later individually identified, allowing us to accurately estimate the number of cats on campus. During the free-ranging cat sampling, we also collected the number of points in which cat food was distributed along and around the transect. All cat-feeding points less than 10 m apart were later considered as single food-source areas.

### 2.3. Historical Records of Red-legged Seriema at Luiz de Queiroz Campus

Between December 2018 and April 2019, we conducted an investigation into the historical occurrence of Seriema in the urbanized area of the LQ campus. We looked through records of old field notes from Alexandrino et al. [8] and personal field notes from 2013 up to 2018. We also conducted unstructured interviews with 18 university members (i.e., professors, researchers, employees, old students) who have been present at the university for 5–20 years. Our intention was to identify a brief past history of Seriema occurrence throughout the campus, find records of past breeding activity, and the occurrence of intentional human food provisions for this species. We also checked bird records in eBird lists and WikiAves (i.e., a Brazilian website for sharing ornithological records, [42]) and in published academic studies and grey literature (e.g., unpublished monographs, congress abstracts).

### 2.4. Monitoring of Red-legged Seriema Individuals

Red-legged Seriema shows no sexual dimorphism (i.e., plumage is predominantly gray and beak and long legs are reddish in both sexes) or clear differences among individuals, but the species is easily recognizable by any citizen. Because of these characteristics, we decided to mark an individual and use a collaborative citizen science scheme to implement the monitoring of an individual bird (e.g., [43]). On the 5th November 2018, we captured and marked one Seriema fledging with a numbered metallic band provided by the Centre for Bird Monitoring of the Brazilian Institute for Environmental Affairs (CEMAVE/IBAMA). The capture occurred next to our laboratory (i.e., LEMaC Wildlife Ecology, Management and Conservation Lab, see Figure S1). The fledging was walking alone with no sign of other accompanying individuals. All marking procedures occurred in less than five minutes and the individual (hereafter banded fledging) was released in the same location. By doing further investigations among university members (i.e., students, professors, faculties staff, visitors) and on the WikiAves website, we concluded that this individual hatched around the 5th and 9th October 2018, in a nest close to the university’s main building and beside a large grassland used for human leisure within the urban area of the campus (i.e., 450 m away from our laboratory). Two fledglings were born in that nest on that occasion [44], so the fledging was around 27-days-old when captured. The nest was 5 m above the ground [45] in the fifth fork of a *Tipuana tipu* tree, which was approximately 15 m tall and with a chest height circumference of 2.10 m [46].

On the morning of November 6th, we were informed that the banded fledgling had joined up with family members (i.e., one parent and its sibling). Parental care of Seriema fledglings usually continues for 7 or 8 months after hatching [27,45,47]. Thus, that afternoon, we used social media (such as Facebook pages published by LEMaC and various ESALQ student and staff groups, as well as individual pages) and internal emails to request campus users to report the location of the banded fledging and its family members on campus during the study period. Our message explained that by marking that individual we had started a long monitoring program of those individuals using records voluntarily provided by citizens (e.g., photographs, videos, short reports). We encouraged citizens to record the day, hour, campus location, and how many Seriemas were present, if the metallic band was visualized on the banded fledging, as well as requesting that any picture or short video should have a brief description regarding observed behavior (using citizen’s own words). All records were sent to us through a Facebook group created for the monitoring or via email. Although our volunteer-based monitoring requested mainly records of the banded bird and its family, records of any other Seriemas within the campus were also welcomed. Given the high-quality of data that was collected, it was possible to differentiate between the family groups and individual members. The GPS coordinates of all records sent were taken by us using Google Earth imagery in decimal degrees and confirmed by the person who collected the data points. Although the volunteer-based monitoring continues, we only considered records sent to us between 6 November 2018 and 4 April 2019 for this manuscript, totaling 149 days of participatory monitoring.

In parallel with the citizen science method, we also did ad libitum field survey to follow the banded fledging and its family, also taking notes about their occurrence and behavior. We conducted six hours of field efforts spread across 29 days during the period mentioned above.

### 2.5. Data Analysis

#### 2.5.1. Red-legged Seriema Behavior Classification

Notes on behaviors exhibited by the Seriemas on the LQ campus were based on Silva et al. [32]. This study described 41 behaviors of the species in a rural landscape within the Cerrado biome in central Brazil, and it is the most complete reference regarding Seriema behavior in a natural habitat. These behaviors are grouped into seven categories, and each includes varieties of specific behaviors. Because our sampling period did not cover the reproductive phase, we skipped five behaviors related to reproduction, and then we used 36 behaviors from six categories: resting, locomotion, ingest/excretory, comfort/maintenance, social behavior, vocalization, (see details in [32], but see Table 2 in the results section). To do so, we used records from Seriema individuals that were observed in the urbanized area of the campus. However, we only used those photograph or video records that included a description of what the volunteer had recorded, and those without this information were only considered if the record itself could provide a clear indication of the behavior that had occurred. When possible, we also contacted the volunteer to get further information about the record.

Because there are no studies reporting Seriema in contact with humans in urban areas (i.e., with exception of those cases on captivity or domesticated), we also described new behaviors related to anthropogenic factors. To do this, we used the same data recorded for the natural behavior classification. Firstly, we listed all interactions with anthropogenic factors observed from the records, of which there were 13 specific behaviors (Table 1). Hence, we grouped them as four categories of general behaviors: interaction with cats, interactions with urban structures, feeding on human-provided food, and exposition to car traffic.

#### 2.5.2. Spatial Analysis: Red-legged Seriema, Cats, and Cat-feeding Points

We used density statistics [48] to explore the use of space and density of Seriemas (i.e., the family of the banded fledging, and another identified individual named ID4, see results) and cats. We also used density statistics for cat-feeding points to explore the spatial distribution and density of food sources. In doing so, we projected the record points expressed as Universal Transverse Mercator (UTM, Datum WGS 84, Zone 23) and calculated densities for each data group. We used the same geographic records to elaborate the polygons of distributions based on the convex hull approach [49]. Thus, we obtained the spatial overlap between target-groups (i.e., family, ID4, cats, and cat-feeding points). We performed all spatial analyses using R [50] based on maptools [51], sp [52], and rgdal [53] packages.

Because our volunteer-based monitoring approach could be considered biased, favoring the collection of records of Seriemas in areas most frequented by citizens, and was dependent on the citizen’s willingness to deliver the record to us, we conducted a pragmatic estimation of the monitored family home-range using the convex hull approach and manual linkage of the extreme occurrence points followed by area calculation (through ArcGIS; [54]).

## 3. Results

### 3.1. Red-legged Seriema, Cats, and the Provision of Cat Food

After 86 h of fieldwork in 2017, we observed 1516 encounters with cats, corresponding to 17.6 cat encounters per sampling hour. From this total, 1119 cat encounters were obtained for samplings conducted during the evening, while 397 were for samplings conducted during the day. We identified 245 cats in the urbanized area. We counted 351 cat-feeding points, which corresponded to 35 different food-source areas. Cat density overlapped 98.8% of the occurrence of the cat-feeding points. However, during the 2018–2019 Seriema monitoring, we also identified six new source areas for cat food (Figure S1). Considering this, altogether the food-source areas were on average 71.5 m apart (minimum distance = 11.03 m, maximum distance = 236.4 m).

**Figure 2:**
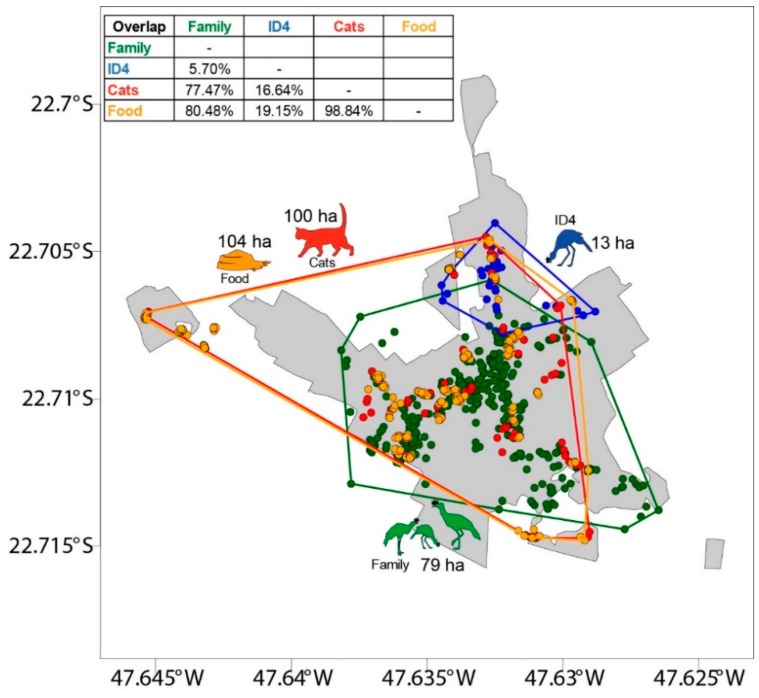
Polygons of distribution based on the convex hull approach showing the home-range size and spatial overlap between: Red-legged Seriema family (green), Seriema individual “ID4” (blue), free-ranging cats (red), and cat-feeding points (orange), within the urbanized area of the “Luiz de Queiroz” campus. Citizen’s records were used to estimate the home range of the Seriemas.

During the whole period of our participatory monitoring, we obtained 401 records of Seriema from 122 different days. Most of the records were provided by the 137 volunteers (i.e., 329 records, while the remaining 72 were provided by our team), and they provided the highest number of the days of monitoring (i.e., records were from 114 days, while we added only eight new days in the monitoring season). Assuming the volunteers spent on average three minutes for each recording (i.e., noticing the bird, recording by photograph or writing, sending the record to us) we calculated that 16 h and 27 min of fieldwork was achieved by volunteers. Thus, summing volunteers’ effort with our six hours of sampling, we ended up with 22 h and 27 min of monitoring effort. Of the 401 records, 365 were exclusively from the banded individual or its family members. From our monitoring scheme we concluded that 28 of the records were from another individual, which we named as ID4. Four records were assumed to be other individuals, due to the distance of their records from the monitored family and the ID4. We also received three records about antagonistic encounters between the monitored family and ID4 (Figure 2, Figure S1).

Approximately 97% of the records (n = 353) of the banded fledging and its family were inside the urban area. Considering the territorial behavior of Seriema [55] and the records of the other individuals surrounding the monitored family (Figure S1), we estimated this family’s territory to be between 53.26 ha (i.e., manual estimation by linking the extreme occurrence points) and 79 ha (i.e., through the convex hull approach) (Figure 2).

**Figure 3:**
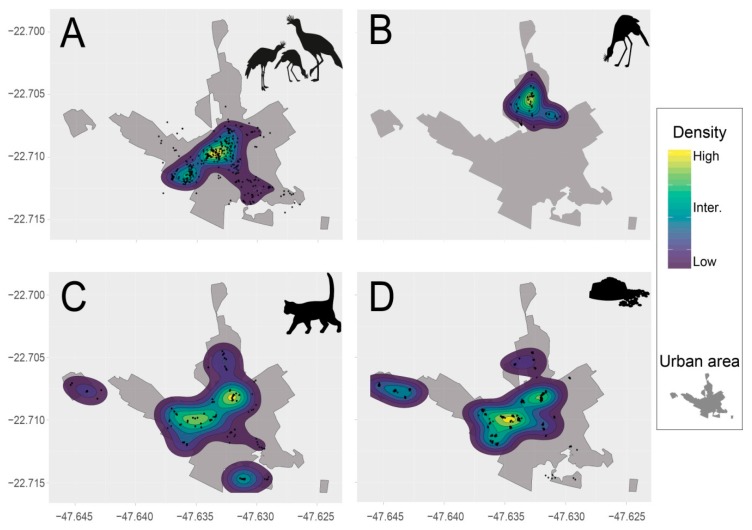
Multi-state ordered density analysis showing the distribution and density of: (**A**) monitored Red-legged Seriema family, (**B**) Seriema individual “ID4”, (**C**) domestic cats, and (**D**) cat-feeding points, within the “Luiz de Queiroz” campus.

The results showed a clear segregation between the area used by the banded fledging and its family and the area used by ID4. However, all of these individuals had a higher density of observations in the urban areas (Figure 3, Figure S1). As expected, cat density was much higher closer to the cat-feeding points. The density of Seriema records also overlapped with cat density and the cat-feeding points (Figure 2 and Figure 3). The monitored family overlapped 80.48% with the cat-feeding points, and consequently also overlapped 77.47% with cat occurrence. On the other hand, the spatial overlap between the Seriema family and the ID4 was <6% (Figure 2).

Our review and interviews indicated that Seriemas have been sporadically recorded in the urbanized zone of the campus since the early 1990’s (Figure 1, Table S1). Six respondents confirmed that nests and fledglings were recorded in the urbanized area before 2018. Five of them had seen fledglings and adults walking in this area approximately at the end of 2016/beginning of 2017, but just one responder had seen breeding activity in 2008 or 2009.

### 3.2. Red-legged Seriema Behaviors

From the 401 records of Seriema, 273 were useful for classifying natural behaviors. We registered 29 different natural behaviors in the urbanized area of the campus. Only reproduction-related behaviors were not observed. Behaviors related to resting and locomotion were the most registered (Table 2), but these results were biased by the participatory monitoring approach, and precaution is necessary for any interpretation (see discussion). For example, full vocalization by the Seriema is mainly done during the first hours in the morning and is extremely loud [27,45]. Few citizens are present in the campus in the early morning, which resulted in few records of this behavior.

**Figure 4:**
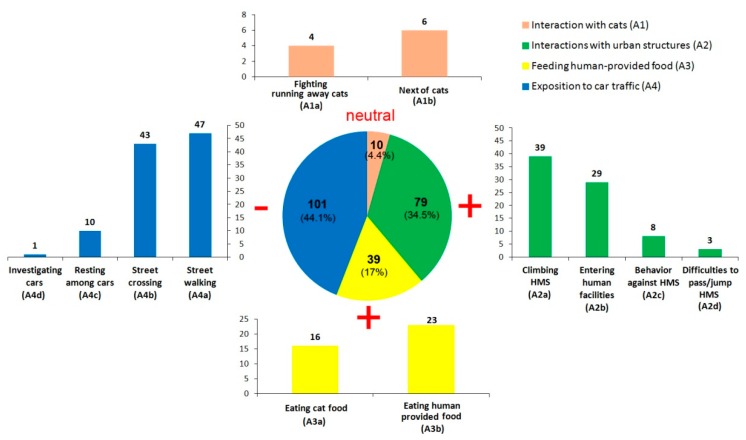
Quantification of the behavior of the Red-legged Seriema related to anthropogenic factors that were observed in the urban area of the “Luiz de Queiroz” campus. In all graphs, the *Y*-axis displays the absolute number of each behavior. The signs show whether general behaviors are helping (+) or harming (−) the Seriemas to adapt to urbanized zones. Some behaviors are considered neutral. Acronyms are: HMS = human-made structure. For a detailed description of each behavior see Table 1.

One hundred and ninety records (i.e., 47.3% of the 401 records) were Seriemas involved in some behavior related to anthropogenic factors in the urban area. Exposure to car traffic was the most common (44.1% of the 190 records), where Seriema were caught calmly walking or crossing streets or resting next to parked cars (Figure 4). Among the interactions with urban structures, Seriema were recorded climbing walls, gates, and low-rise buildings to perform vocalizations or observations from the top. Seriemas were also observed entering human facilities, such as laboratories and class buildings. The monitored family was observed more often whilst eating human food left for them intentionally, than cat food. Few negative interactions with cats were observed.

We observed the banded fledging having difficulties overcoming 1.8 cm-high wire fences on three occasions. The first was when the fledgling was +/− 27 days old, on the same day as we did the capture and marking. The second occasion was in mid-December 2018 when it was +/− 80 days old. The fledging had already grown its flight feathers and had been observed doing short flights. However, the fledging did not use its wings to cross the barrier. The last time was when the fledgling was +/− 122 days old (19th of January 2019). On this occasion, its sibling and parent quickly overcame the wire fence by jumping while flapping their wings. Before the banded fledgling did the same, it walked back and forth along the fence for several minutes.

On the 13th of February 2019, the non-banded fledgling was hit by a car and died days after. After this incident, all records from the family were about the banded fledgling and its parents.

## 4. Discussion

Understanding the implications of the human-modified landscapes on biota is paramount for modern ecologists. Empirical evidence suggests that the vast majority of species, especially large- vertebrates, do not prosper in highly modified and fragmented landscapes [56,57,58]. However, the results of our study show that this large bird species exhibits some tolerance to urbanized zones with high human presence. This suggests behavioral adaptation that leads to a non-random change in their survivorship, also indicating a process of non-intentional domestication. The monitored individuals of Seriema shared the spatial resource with free-ranging cats, interacted with a variety of human-made structures, and were exposed to threats from automotive traffic. Analyzing the interactions between this bird and each factor indicates the presence of behaviors that both increase survival and cause detrimental impacts. Presumably, existing in this environment offers both benefits (e.g., absence of an apex-predator, flexibility of the foraging strategy, improved territorial defense by using human-made structures, decrease of intra- and inter-specific competition) and costs (e.g., early mortality due to physical risks).

### 4.1. Space Sharing between Red-legged Seriema, Cats, and Humans

The monitored Seriema family occupied an estimated urban area up to 79 ha, a home range higher than the size observed for groups of Seriema in a non-urbanized landscape [55]. More than 75% of the family home range was shared with free-ranging cats, which was tied to the distribution of cat-feeding points. Few negative interactions between Seriemas and cats were reported, likely at least in part to the fact that these species have distinct activity periods (i.e., cats had higher activity during the evening [59], Seriemas are diurnal [27]). Although free-ranging cats can cause high rates of wildlife mortality [21,22] the size, shape, and body mass of an adult Seriema makes predation from cats unlikely, however, young fledglings may be vulnerable [21,22]. During interviews, one respondent mentioned that they believed to have seen a Seriema fledgling being attacked by a cat in the past, although few details were provided. Thus, based on our current data, sharing space with free-ranging cats seems to be a neutral factor with regards to population fitness (neither detrimental nor beneficial) and has little influence on patterns in behavior for the Seriemas (Figure 4). However, the presence of cat-feeding points, supplied with food on a daily basis by local residents, provide a consistent food source for both juvenile and adult Seriemas. Given that Seriemas are omnivorous, the available dried cat food from the cat-feeding points provide a suitable supplement for much of their nutritional requirements [47,60,61]. This indirect benefit is likely to positively influence population fitness of Seriema in this urban area (Figure 4), and the wide area across which the food is provided may explain the large home range. However, this behavior, which favored areas with cat-feeding points, may lead these birds to become dependent on anthropogenic food, consequently leading to non-intentional domestication [13]. Indeed, data from interviews show examples of citizens intentionally feeding Seriemas, and the review of older data revealed that this activity is nothing new.

All records of Seriemas using human-made structures did not show evidence of negative reaction by campus users. People often tend to become more aware of the surrounding environment when they are close to nature [62] and in recent years in Brazil activities such as birdwatching have become increasingly mainstream (e.g., [42,43]), which suggests that people are becoming more positive toward wildlife in general [63,64]. Indeed, the willingness of many citizens to participate in our study supports this assumption. Although man-made structures may act as barriers for small-bodied birds [7,16,17], the Seriema may benefit from the availability of some structures of which they can utilize to demonstrate their natural behaviors, such as performing territory defense displays on top of fences, statues, or house roofs [55]. Vocalizations used in territorial defense may be heard over long distances and this may be increased when performed on top of tall structures [27,45,65]. Seriemas also tended to frequent human structures where food provisions were available, either at cat-feeding points or where people directly offered food to the birds. This tendency may provide positive encounters between birds and humans and perpetuate this behavior [66], thus increasing the chances that these individuals will persist in this urbanized area.

Although it appears that humans provide benefits for the Seriemas, car traffic on the LQ campus presents a major threat to their survival [20,25,66]. Being able to perceive the associated risks and avoiding cars [67] is an important adaptation for species inhabiting urbanized zones [25]. However, the incident which resulted in the death of a juvenile after being hit by a car within the study (Figure 4) shows that cars are a continuous threat to these birds. Cases of Seriema roadkill in peri-urban areas have been previously reported (e.g., [18,19]), which suggests that the species may have difficulties in perceiving the risk associated with cars.

Territorial behavior was present between the Seriemas on the urban area of the campus. The monitored family avoided the individual ID4, sharing less than 6% of space, and agonistic encounters were observed, indicating intraspecific competition for space, and probably for the food resources (provided by humans or not). During monitoring, other individuals from outside groups were not recorded in the family’s territory or in ID4’s territory (Figure S1). Few territory overlaps were also observed by Souza et al. [55], whilst antagonistic interactions relating to territory defense were reported. Thus, considering that few interactions were recorded involving Seriema and other species (such as domestic cats), intraspecific competition seems more important than interspecific competition. Due to this antagonistic interaction, the individual ID4 and free-ranging cats had only 16.7% of space-use overlap. We provide a note of caution about the possible bias in estimation of ID4’s space use, because the records were provided only from places with human presence.

### 4.2. Red-legged Seriema Adaptation to Urbanization

Our review on historical records of Seriema confirms that the species arrived in the urban area of the LQ campus more than 20 years ago, but records of a fledgling are more recent. Because our method of investigation accessed limited information from the past, we are not sure if the species is still in the process of establishment in the urban area of the campus or is actually in the increasing stage, following definitions specified in Sol et al. [25].

Our results pointed out that the species have adapted to finding food in the urban area (i.e., capturing natural prey, or accepting human food directly or indirectly provided) and avoiding conflicts with free-ranging cats for food. However, this large bird is still frequently exposed to car traffic, which is a threat to individual survival. Thus, this behavior makes us question if the species is still in the process of acquiring useful learned behaviors to survive in this ecosystem [25].

Some individual’s personalities may present risky behaviors that threaten their fitness [68]. We did not find information on what happened with the fledglings born in the years 2009 and 2016. If they were able to avoid threats from cars and the car collision that we reported was just an isolated case, it may suggest that the personality of that non-banded juvenile individual may have contributed to the accident. Then, by this other point of view, we would accept that the species is actually in the increasing stage, as they have already developed all behaviors to avoid mortality risk [25]. The sporadic occurrences of the monitored family in a more urbanized area outside the limits of the campus would support this assumption (Figure S1, see point at south). However, it may be premature in assuming this. In recent years, the landscape surrounding the LQ campus has witnessed significant urban intensification [8,38]. This may have limited the available space for the dispersal of individuals born in the campus and may indicate that individuals are actually using urban areas as last resort, even if all necessary behavior to survive is lacking.

### 4.3. Red-legged Seriema and Free-ranging Cats—Future Conflicts?

Although our results show Seriemas have benefited from food provisions for cats, if cat populations continue to increase on the LQ campus, the chances of negative interactions with Seriema would also increase, such as injuries caused by conflict for food or predation of juveniles. Also, as cat-feeding points are mainly located along the sidewalks of streets, the tendency of Seriemas to frequent these areas may expose them to greater risks of car collisions. Consequently, with cats becoming an accepted part of the landscape, it is expected that the implementation of cat-control programs would become increasingly less popular in the future [69,70].

### 4.4. Individual Bird Monitoring Based on Citizen Science in an Urban Ecosystem

Engaging volunteers for fine monitoring in the field (i.e., wildlife individuals) is not a trivial task [71,72], and if not well planned, the risk of obtaining few records from the focus bird increases [43]. Monitoring based on volunteers totaled approximately 22 h and 27 min of sampling effort in five months, while in a traditional monitoring program of behavior ecology, counting with few trained observers, the same effort is reached only after a longer period (e.g., one year in [32]). Through our methodology, we registered 29 natural behaviors from those 36 described in Silva et al. [32] in a rural landscape. Furthermore, although our monitoring based on volunteers has limitations for precise estimations for species home range and behavior quantifications, we achieved a high number of records which add credibility to our results and discussions. In green areas of urbanized zones with high environmental appeal for locals, people easily develop curiosity about the local wildlife [63,64,70]. This is a useful prerequisite for a promising citizen science project engaged in collecting a variety of data from the wildlife in urbanized zones.

## 5. Conclusions

Our hypotheses outlined were supported. The red-legged Seriema is actually able to live in the urbanized zone of the LQ campus, mainly due to human actions that increase their chances of survival, such as direct and indirect food provisioning (i.e., humans offering food for cats). Although the Seriema are interacting with free-ranging cats, the intraspecific competition seems more important than interspecific competition to define spatial distribution of the Seriema in the urban area of the campus. The human contact is also promoting a non-intentional domestication process of Seriemas, which contributes to losing the fear of humans. We also suggest that the Seriemas on the urban area of the campus are in the process of acquiring adaptive behavior because car avoidance is still absent in some individuals.

Although our study is the first case reported in a medium-sized city within the natural geographical distribution of Seriemas, if the same human actions reported here were done in another similar urban area, we believe the same processes may occur.

## Figures and Tables

**Table 1:** Description of behaviors of Red-legged Seriema related to anthropogenic factors that were observed in the Luiz de Queiroz campus.

General Behavior	Specific Behavior	
	Code	Description
Interaction with cats (A1)	A1a	Fighting with cats or running away from them.
	A1b	Staying next to cats but without negative or positive interaction.
Interactions with urban structures (A2)	A2a	Climbing human-made structures (e.g., buildings, walls, poles, etc.) and performing other natural behaviors up there.
	A2b	Entering (or trying) into human facilities (e.g., buildings and other walled/gated spaces)
	A2c	Agonistic behavior against human-made structures (e.g., fighting/playing with a pole, interacting with own reflection in windows or mirrors).
	A2d	Difficulties to pass through or jump human-made structures (e.g., fences, gates, walls).
Feeding human-provided food (A3)	A3a	Eating cat food provided by humans.
	A3b	Consuming any other food provided intentionally or not by humans (e.g., waste, bread, fruits, water from the tap).
Exposition to car traffic (A4)	A4a	Calmly walking in the streets.
	A4b	Crossing the street, walking in front of, or through, cars in movement.
	A4c	Resting among parked cars or below them.
	A4d	Investigating parked cars.

**Table 2:** Quantification of the occurrence of each natural behavior of the Red-legged Seriema in the study area. Behaviors follow Silva et al. [32] where more details on each behavior may be obtained.

Behavior Category	n.	%	Specific Behavior		n.	%
			Code	Description		
Resting (1)	173	33.2	**1a**	Observing	152	29.2
			1b	Resting	21	4.0
			1c	Sleeping in the nest		
			1d	Sleeping on a branch		
			1e	Hiding		
Locomotion (2)	221	42.4	2a	Walking	190	36.5
			2b	Short flight	12	2.3
			2c	Long flight	4	0.8
			2d	Short run	8	1.5
			2e	Long run	2	0.4
			2f	Climbing on a branch	5	1.0
Ingest/excretory (3)	72	13.8	3a	Drinking	7	1.3
			3b	Eating	62	11.9
			3c	Eating crouched	2	0.4
			3d	Defecating	1	0.2
Comfort/maintenance (4)	21	4.0	4a	Preening the chest feathers	3	0.6
			4b	Preening the wing feathers	4	0.8
			4c	Preening the thigh feathers	2	0.4
			4d	Preening the tail		
			4e	Preening the cloaca		
			4f	Preening the dorsum	2	0.4
			4g	Preening the abdomen		
			4h	Dust bathing	1	0.2
			4i	Scratching the head	1	0.2
			4j	Scratching the neck	1	0.2
			4k	Scratching the beak		
			4l	Ruffling	5	1.0
			4m	Repositioning the wings	1	0.2
			4n	Stretching	1	0.2
Social behavior (5)	16	3.1	5a	Agonistic interspecific interaction	4	0.8
			5b	Agonistic intraspecific interaction	5	1.0
			5c	Air contact	3	0.6
			5d	Juvenile chasing	4	0.8
Vocalization (6)	19	3.6	6a	Short vocalization	1	0.2
			6b	Agonistic vocalization	4	0.7
			6c	Full vocalization	14	2.7
TOTAL		522			522	

## Supplementary Materials

The following are available online at https://www.mdpi.com/2076-2615/9/6/351/s1, Figure S1 and Table S1.
