# Supplementary material for: Large Terrestrial Bird Adapting Behavior in an Urbanized Zone
Source: Animals (Basel). 2019 Jun 13;9(6):351. doi: 10.3390/ani9060351 (PMC6617044; doi:10.3390/ani9060351)
Supplement: Supplementary file 1 [file animals-09-00351-s001.pdf]

Large terrestrial bird adapting behavior in an urbanized zone

Supplementary material.

Figure S1 – Spatial distribution of all records of Red-legged Seriema obtained with the participatory monitoring done in the Luiz de Queiroz campus between November/2018 and April/2019. During this monitoring new cat-feeding points were discovered.

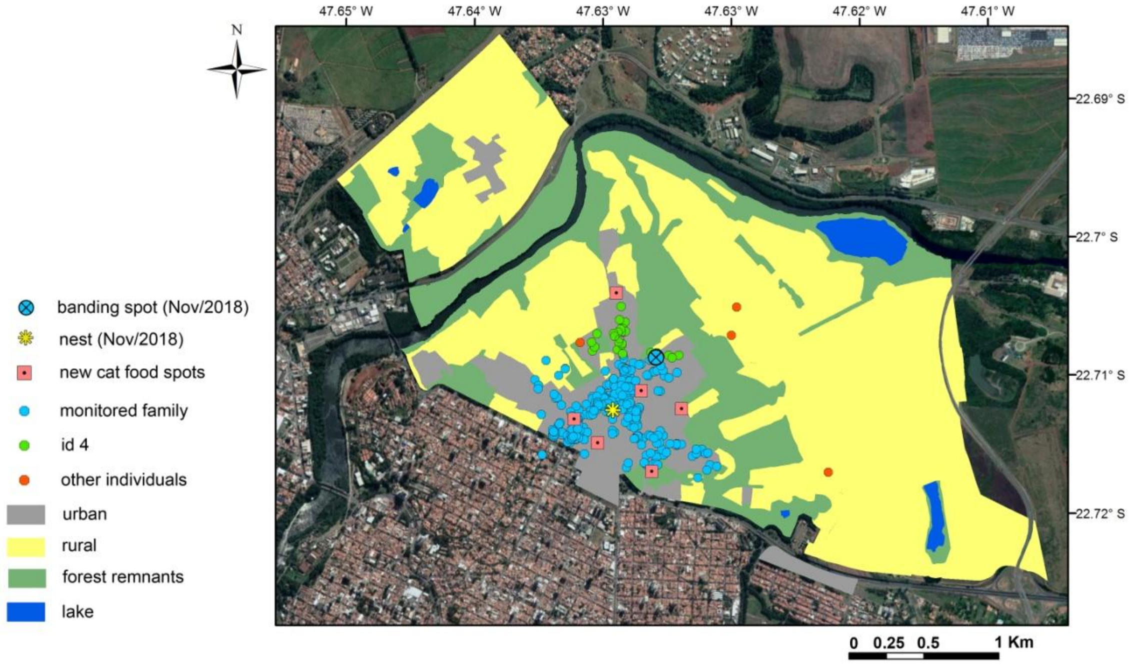

Table S1. Records of Red-legged Seriema obtained before 2018 in the university campus "Luiz de Queiroz", Piracicaba, Brazil. Note: Function = relationship of each author with the university;

Record's author	function	Record type	Source	Record´s year	Strata	Record of reprocticion activity?	GSP location provided?
Eduardo Alexandrino	researcher	our records	unpublished data	2011	urban	no	yes
Eduardo Alexandrino	researcher	our records	unpublished data	2017	urban	no	yes
Juan Domini	student	our records	unpublished data	2017	urban	no	yes
Katia Ferraz	professor	our records	unpublished data	2016	urban	no	yes
Katia Ferraz	professor	our records	unpublished data	2016	urban	no	yes
Katia Ferraz	professor	our records	unpublished data	2016	urban	yes, adult + fledgling	yes
Katia Ferraz	professor	our records	unpublished data	2017	urban	no	yes
Katia Ferraz	professor	our records	unpublished data	2018	urban	no	yes
Katia Ferraz	professor	our records	unpublished data	2018	urban	no	yes
Silvio Marchini	researcher	interview	unpublished data	2014	urban	no	yes
Ana Rita Aleoni	staff	Interview	unpublished data	2003	urban	no	yes
Ana Rita Aleoni	staff	Interview	unpublished data	2016 or 2017	urban	yes, adult + fledgling	yes
Ana Rita Aleoni	staff	Interview	unpublished data	2016 or 2017	urban	no	yes
Ana Rita Aleoni	staff	Interview	unpublished data	2017	urban	no	yes
Celestino Alves Ferreira	staff	Interview	unpublished data	1995	rural	no	yes
Celestino Alves Ferreira	staff	Interview	unpublished data	before 2009	urban	no	yes
Celestino Alves Ferreira	staff	Interview	unpublished data	before 2009	urban	no	yes
Celestino Alves Ferreira	staff	Interview	unpublished data	before 2009	urban	yes, fledgling	no
Celestino Alves Ferreira	staff	Interview	unpublished data	2016 or 2017	urban	yes, adult + fledgling	yes
Conceição Aparecida Dias Ferraz	staff	Interview	unpublished data	2016 or 2017	urban	no	yes
Edno Aparecido	staff	Interview	unpublished data	2015 or 2016 or 2017	urban	no	yes
Francisco José Mandro	staff	Interview	unpublished data	2015 or 2016 or 2017	urban	no	yes
Joao Angelo Cerignoni	staff	Interview	unpublished data	after 1995	urban	no	yes
Joao Angelo Cerignoni	staff	Interview	unpublished data	2017	urban	no	yes
Wilson Vanderlei Jacinto	staff	Interview	unpublished data	before 2009	urban	no	yes
Luiz Carlos Gusmão	staff	Interview	unpublished data	2016 or 2017	urban	no	yes
Ronald Guidotti Filho	staff	Interview	unpublished data	2016	urban	no	yes
Ronald Guidotti Filho	staff	Interview	unpublished data	2016 or 2017	urban	yes, adult + fledgling	yes
Sandra Maria de Mello Lazari	staff	Interview	unpublished data	2016 or 2017	urban	yes, adult + fledgling	no
Sandra Maria de Mello Lazari	staff	Interview	unpublished data	2016 or 2017	urban	no	yes
Sônia Conceição Vicentin	staff	Interview	unpublished data	2016 or 2017	urban	no	yes
José Geraldo Gomes	staff	Interview	unpublished data	1994 or 1995	urban	no	yes
José Geraldo Gomes	staff	Interview	unpublished data	2008 or 2009	urban	yes, fledgling	yes
Danilo Romero	student	Interview	unpublished data	2016	urban	no	yes
Isabela Guardia	student	Interview	unpublished data	2014	urban	no	yes
Magê Deunrago	student	Interview	unpublished data	2012	urban	no	yes
Magê Deunrago	student	Interview	unpublished data	2014	urban	no	yes
Maristela Camolesi	student	Interview	unpublished data	2014	urban	no	yes
Viviane Paulenas	student	Interview	unpublished data	2016	urban	no	yes
Viviane Paulenas	student	Interview	unpublished data	2016	urban	no	yes
Viviane Paulenas	student	Interview	unpublished data	2016	urban	yes, adult + fledgling	yes
André Guaraldo/ Carlos Gussoni	visitor	eBird list	https://ebird.org/brasil/view/checklist/S34775000	2017	rural	yes, adult + nest	no
Alex Bovo	student	WikiAves	https://www.wikiaves.com.br/955351	2013	rural	no	yes
André Araújo	visitor	WikiAves	https://www.wikiaves.com.br/120371	2010	?	no	no
Edinaldo Barbosa	visitor	WikiAves	https://www.wikiaves.com.br/381714	2011	?	no	no
Antonio Vallese	visitor	WikiAves	https://www.wikiaves.com.br/2833206	2017	urban	no	yes
Francisco T. Tatit	visitor	WikiAves	https://www.wikiaves.com.br/2154242	2016	urban	couple	no
Henri Coronfly	visitor	WikiAves	http://www.wikiaves.com/2242738	2016	urban	no	no
José Libório	visitor	WikiAves	https://www.wikiaves.com.br/2153091	2016	urban	couple	no
José Libório	visitor	WikiAves	https://www.wikiaves.com.br/1484149	2014	urban	no	no
José Luís da Cruz	visitor	WikiAves	https://www.wikiaves.com.br/1183847	2013	urban	no	yes
Luciana Silva	visitor	WikiAves	https://www.wikiaves.com.br/1586504	2015	urban	no	yes
Luis Fabiano Ortiz	visitor	WikiAves	https://www.wikiaves.com.br/1593694	2015	urban	no	yes
Marcos Siqueira	visitor	WikiAves	https://www.wikiaves.com.br/931882	2013	urban	no	yes
Naydja Maimone	visitor	WikiAves	https://www.wikiaves.com.br/1550233	2014	urban	no	yes
Vittor Miranda	visitor	WikiAves	https://www.wikiaves.com.br/1588398	2015	urban	no	yes
Eduardo Alexandrino	researcher	literature	Alexandrino et al. 2013	2008	rural	no	no
Eduardo Alexandrino	researcher	literature	Alexandrino et al. 2013	2010	rural	no	yes
Eduardo Alexandrino/Alex Bovo	researcher	literature	Alexandrino et al. 2013	2010	rural	no	yes
Eduardo Alexandrino/Daniela Luz/Alex Bovo	researcher	literature	Alexandrino et al. 2013	2008	rural	no	yes
Eduardo Alexandrino/Julio Cesar Costa	researcher	literature	Alexandrino et al. 2013	2004	rural	no	yes

Eduardo Alexandrino/Paulo	researcher	literature	Alexandrino et al. 2013	2007	rural	no	yes
Julio Cesar Costa	student	literature	Alexandrino et al. 2013	2004	rural	no	yes
Julio Cesar Costa	student	literature	Alexandrino et al. 2013	2004	rural	no	no
Julio Cesar Costa	student	literature	Alexandrino et al. 2013	2004	rural	no	no
Julio Cesar Costa	student	literature	Alexandrino et al. 2013	2005	rural	no	yes
Julio Cesar Costa	student	literature	Alexandrino et al. 2013	2005	rural	no	no
Julio Cesar Costa	student	literature	Alexandrino et al. 2013	2005	rural	no	yes
Julio Cesar Costa/Gustavo Betini	student	literature	Alexandrino et al. 2013	2002	rural	no	no
Julio Cesar Costa/Gustavo Betini	student	literature	Alexandrino et al. 2013	2002	rural	no	no
Julio Cesar Costa/Gustavo Betini	student	literature	Alexandrino et al. 2013	2002	rural	no	no
Julio Cesar Costa/Gustavo Betini	student	literature	Alexandrino et al. 2013	2003	rural	no	yes
Julio Cesar Costa/Gustavo Betini	student	literature	Alexandrino et al. 2013	2003	rural	no	no
Eduardo Alexandrino	student	literature	Alexandrino et al. 2013	2011	urban	no	yes
Gustavo Betini	student	literature	Alexandrino et al. 2013	1996	urban	no	no
